# A comparison of inpatient admissions in 2012 from two European countries

**DOI:** 10.7448/IAS.17.4.19712

**Published:** 2014-11-02

**Authors:** Victoria Tittle, Giovanni Cenderello, Ambra Pasa, Preya Patel, Stefania Artioli, Chiara Dentone, Paolo Fraccaro, Mauro Giacomini, Maurizio Setti, Antonio Di Biagio, Mark Nelson

**Affiliations:** 1 HIV Unit, Chelsea and Westminster Hospital, London, UK; 2 Infectious Diseases Unit, EO Ospedali Galliera, Genoa, Italy; 3 IT Department, EO Ospedali Galliera, Genoa, Italy; 4 Infectious Diseases Unit, ASL-5 La Spezia, La Spezia, Italy; 5 Infectious Diseases Unit, ASL-1 Imperiese, Sanremo, Italy; 6 Centre for Health Informatics, University of Manchester, Manchester, UK; 7 Biomedics Engineering, University of Genoa, Genoa, Italy; 8 Immunology Unit, San Martino Hospital/University of Genoa, Genoa, Italy; 9 Infectious Diseases Unit, San Martino Hospital/University of Genoa, Genoa, Italy

## Abstract

**Introduction:**

This study compares the trends of HIV inpatient admissions between a London tertiary HIV centre (United Kingdom) and four infectious disease wards in Italy (IT) to recognize common patterns across Europe.

**Methods:**

Data regarding HIV inpatient admissions was collected by using discharge diagnostic codes from 1 January to 31 December 2012, including patient demographics, combined antiretroviral therapy (cART) history, CD4, viral load (VL) and mortality rates. Discharge diagnoses were categorized according to the International Classification of Disease (ICD) 9 and 10 system. All ICD categories that reach a 3% threshold of total admissions were analyzed.

**Results:**

A total of 731 admissions (257 in Italy and 474 in the United Kingdom) for 521 patients (1.5 mean admission per patient). Female admissions were higher in Italy at 22.6% (n=58) compared to 14.9% (n=47) in the United Kingdom. Median age of patients was 47 years old. There was an undetectable VL in 65.8% (n=169) of admissions in Italy and 67.1% (n=319) in the United Kingdom (p=0.385); 86.4% (n=222) and 82.4% (n=389) of admissions were on cART, respectively. Mean CD4 was 302 in Italy compared to 368 in the United Kingdom (p=0.003). Average length of admission was 16 days with a 10.2% (n=21) mortality rate in Italy compared to 8 days with 2.8% (n=9) mortality in the United Kingdom (p<0.001). HCV co-infection was present in 64.6% (n=166) in Italy and 13.5% (n=64) in the United Kingdom and commonest mode of transmission was needle use in Italy (67.3%, n=173) and men who have sex with men in the UK cohort (59.9%, n=284). The cause of inpatient admissions according to ICD codes can be seen in following Figure 1.

**Conclusions:**

Significant differences in the duration of inpatient admission and mortality rates can be observed between these two cohorts which is secondary to the impact of Hepatitis C co-infection in Italy. However increases in the number of Hepatitis C co-infection patients amongst MSM in London has been reported [1] and route of transmission in Italy is shifting towards MSM [2], therefore it is important to learn how HIV is developing and managed in a global context to help plan future for services. The UK cohort demonstrates a wider range of conditions necessitating admission, and with an ageing HIV population, this is expected to increase in the future, requiring general and specialist HIV physicians to work closely together. The HIV-RNA threshold is 400 copies/mL to account for blips according to British HIV Association (BHIVA) Guidelines 2012 [3].

**Figure 1:**
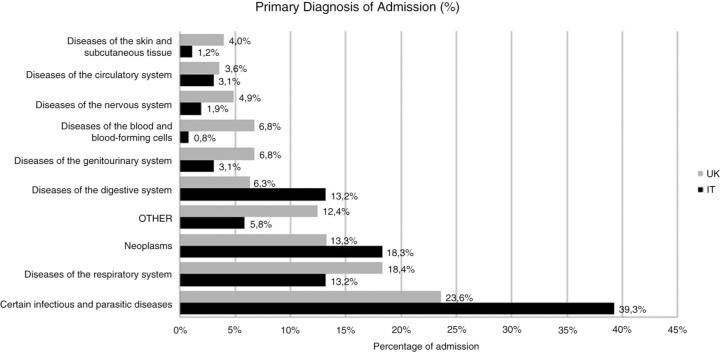
Primary diagnosis admissions according to ICD classification (%) in the two cohorts.
